# Management of sexually transmitted infections: a qualitative assessment of community pharmacy practices in the Ho Municipality, Ghana

**DOI:** 10.1186/s40545-023-00650-0

**Published:** 2023-11-10

**Authors:** Araba Ata Hutton-Nyameaye, Farrukh Ishaque Saah, Israel Bedzina, Samuel Owusu Somuah, Kofi Boamah Mensah, Kwabena Obeng Duedu, Kwame Ohene Buabeng

**Affiliations:** 1 Department of Pharmacy Practice, Faculty of Pharmacy and Pharmaceutical Sciences, Kwame Nkrumah University of Science and Technology, Kumasi, Ghana; 2 Department of Pharmacy Practice, School of Pharmacy, University of Health and Allied Sciences, Ho, Ghana; 3 Department of Epidemiology and Biostatistics, FN Binka School of Public Health, University of Health and Allied Sciences, Hohoe, Ghana; 4 Department of Population and Health, Faculty of Social Sciences, College of Humanities and Legal Studies, University of Cape Coast, Cape Coast, Ghana; 5 Department of Medical Laboratory, School of Allied Health Sciences, University of Health and Allied Sciences, Ho, Ghana; 6 Department of Biomedical Sciences, School of Basic and Biomedical Sciences, University of Health and Allied Sciences, Ho, Ghana; 7 College of Life Sciences, Birmingham City University, City South Campus, Birmingham, UK

**Keywords:** Sexually transmitted infections management, Community pharmacy, Pharmacy staff, Ghana

## Abstract

**Background:**

Effective management of sexually transmitted infections (STIs) is crucial in the control and spread of these infections in health systems. Community pharmacies are usually the first port of call in Ghana for most people who contract STIs for therapy. Delayed and inappropriate treatment contributes significantly to treatment failures, drug resistance and complications. However, the community pharmacies may not have diagnostic tools and trained personnel for prompt case detection and appropriate therapeutic action. Thus, posing a higher risk for inappropriate therapy with consequences of worsening symptoms and poor treatment outcomes. This study explored the STI management practices in community pharmacies in the Ho Municipality.

**Methods:**

Purposively selected study participants were community pharmacy staff including Pharmacists (*n* = 6), Pharmacy Technicians (*n* = 2) and Dispensing Assistants (*n* = 10) in outlets in Ho Municipality of the Volta region, Ghana. Data collection was carried out from December 2020 to January 2021. In-depth interviews of the participants using a semi-structured interview guide were conducted and recorded. Data obtained was transcribed and analyzed using NVivo version 12 using the thematic framework.

**Results:**

Some of the pharmacy staff were unaware of National Standard Treatment Guidelines (STG) and its recommendations for STI management. More than half of the participants believed the STG recommendations were important for therapy but few thought the STG recommendations were ineffective sometimes. Appropriate STI management practices observed included infection treatment based on laboratory data, and STG protocols that recommend syndromic approach. Negative STI management practices included disregarding the presence of possible mixed infections and treating all symptoms observed empirically as a single infection without laboratory confirmation.

**Conclusion:**

The STI management practices in the community pharmacies had many gaps that risk infective therapy, treatment failures, STI complications, and antibiotic resistance. Efforts should be invested into the training of practitioners in community pharmacies for safe and effective practices for STI management, and encouraged to have diagnostic kits or work with laboratory facilities for testing to inform definitive therapy for optimal outcomes.

**Supplementary Information:**

The online version contains supplementary material available at 10.1186/s40545-023-00650-0.

## Introduction

Sexually transmitted infections (STIs) pose a significant public health challenge worldwide. STIs incidence remains high globally, with more than one million daily infections and an estimated 498 million new curable STIs occurring annually [1]. Sub-Sahara Africa is the most affected region worldwide [2] with about 93 million people living with STIs [3]. According to a recent study, the prevalence of STIs in sub-Saharan Africa was approximately 7.7% [4]. Osei-Yeboah et al. also reported that HIV (human immunodeficiency virus) and syphilis prevalence among blood donors in the Ho municipality, Ghana, were 4.78% and 2.5%, respectively [5]. The Ho region recorded the highest prevalence of HIV/AIDS (2.7%) in Ghana in 2017 [6] and currently, 6.03% of its population lives with HIV/AIDS alone [7]. Given the prevalence of STIs, early diagnosis and efficient management are critical and will remain an essential part of STI control programs [8].

It has been reported that delayed and improper treatment practices have led to irreversible drug-resistant infections [1, 9]. STIs prevention and control have widespread health effects, such as a reduction in the transmission of STIs, and prevention of complications such as pelvic inflammatory disease (PID), infertility, foetal death, and congenital infections [1, 9, 10]. The World Health Organisation (WHO) global health sector strategy on STIs, 2016–2021, aims to end STIs as a worldwide epidemic by 2030 [11]. To stop global transmission of STIs and fully eradicate them, the most important goals are to keep people from getting infected and offer supportive services and treatment to affected individuals [11]. Therefore, measures must be implemented to enhance STI case management.

In Ghana, people with STIs can get treatment or advice from a variety of sources, such as public or private health institutions, traditional or herbal doctors, faith healers, and self-medication [2]. Due to a lack of resources, poor healthcare system, and inadequate medical personnel, Ghana's primary healthcare system is not fully able to provide reproductive health services [12, 13]. This gap in the healthcare system has advantageously positioned public or community pharmacies to offer reproductive healthcare services [13, 14]. The Ministry of Health (MOH), Ghana, with support and funding from the National AIDS Control Programme (NACP) and the West Africa Project to Combat AIDS and STD (WAPTCA), have trained community pharmacists in the syndromic management of STIs [15]. The syndrome-case management of STI relies on identifying the syndromes, followed by standardized treatment to deal with the causing organisms, and is straightforward, affordable, and does not require a laboratory test [16]. Studies conducted in Ghana and other nations have demonstrated that community pharmacists provide a wide range of health services in addition to their traditional role of dispensing medication [17, 18].

However, little is known about STI management practices at the community pharmacies post MOH training and that it was not a country-wide community pharmacists training. Therefore, it is essential to explore the knowledge and skills of community pharmacists and their staff on the appropriate management of STIs.

## Methods

### Study setting and design

The research was done in the Ho Municipality, one of the 25 administrative districts in Ghana's Volta Region [19]. Ho serves as both the municipality's and the Volta region's capital, making the municipality the biggest urban area in the region [19]. A recent study indicates that common STIs, such as syphilis, have recorded a prevalence rate of 2.6% in the Ho municipality [5].

This study was a qualitative study which employed exploratory design to conduct in-depth investigation into the management of STIs by community pharmacists and pharmacy support staff from December 2020 to January 2021. This design allowed for qualitative data collection through interviews to gain in-depth knowledge about STI management by community pharmacists and their support staff.

### Population and sampling

In Ghana, the pharmacy staff is mostly made up of the pharmacist, pharmacy technician and dispensing assistants. Pharmacy technicians and dispensing assistants are not pharmacists but are certified by the Pharmacy Council of Ghana to assist the pharmacist in delivering pharmaceutical services. The participants comprised community pharmacists (n = 6), pharmacy technicians (n = 2) and dispensing assistants (n = 10). These are individuals who provide over-the-counter medication services in community pharmacies. They are the first call point of care for most for advice on infection and recommending/prescribing medications to such diagnoses and self-care treatment of minor illnesses [20].

The participants were purposively selected, considering their years of community pharmacy services and experience in providing treatment for people with STI. This was to ensure that only community pharmacists and other pharmacy staff with experience in demonstrating knowledge and practice of STI management.

### Data collection procedures

Data were collected through face-to-face interviews using a semi-structured interview guide. The interview guide was structured into three parts. Part I collected information about participants’ background characteristics, such as demographic and professional information. The second part focused on assessing participants’ knowledge and perception of the national Standard Treatment Guidelines (STG) in managing STIs, whereas the final part explored their STI management practices. Interviews lasted for an average of 15 to 25 min and were tape- recorded with the consent of the participants. Appointments were scheduled with the selected participants at their convenience and choice of place for the interviews. However, most of the interviews took place at the community pharmacy (in the pharmacist’s office), away from the customer waiting area, to allow for privacy, and prevent interruptions and reduce noise interferences. Interviews were conducted in the English language (Additional file 1).

### Ethical considerations

Ethical approval was obtained from the Ghana Health Service Ethics Review Committee (GHS-ERC002/05/20) and the Committee for Human Research, Publication and Ethics-Kwame Nkrumah University of Science and Technology (CHRPE-KNUST) [CHRPE/AP/178/21]. Written informed consent was taken from the participants prior to their inclusion. This was done by explaining the study purpose and procedures to the prospective participants, with those interested in participating affirming their agreement by endorsing the consent form. Anonymity about study participants was maintained by not including any personally identifying information of participants but rather using pseudonyms. Confidentiality was ensured by restricting data collected to only the investigators without access to any third parties.

### Data analysis

All recorded interviews were transcribed verbatim and prepared for analysis by proofreading and revising transcripts with audio recordings. Analysis was conducted using NVivo version 12 using thematic analysis. The analysis was inductive and adopted a six-step thematic analysis framework by Braun and Clarke in carrying out the analysis. These six steps follow as familiarizing with the data, coding the data, creating themes, revising of themes, defining the themes and writing the thematic analysis [21]. Coding was done independently by two of the authors (AAHN and FIS) and where there were disagreements on codes and themes, a third opinion was given by IB. Thematic results were presented in a table, and participant quotes were included to substantiate the themes and codes.

## Results

### Demographic information of study participants

More than 80% of the participants (n = 16) were aged between 21 and 30 years. There was an equal number of females and males (n = 9). 10 participants (> 50%) were dispensing assistants, 6 were community pharmacists, and the remaining 2 were pharmacy technicians. The majority of the participants had 2 to 3 years of working experience in community pharmacy practice (Table 1).

**Table 1:** Demographic characteristics of the participants

Variable	Category	Frequency (%)
Age	21–30	16 (88.9)
	31–40	2 (11.1)
Sex	Male	9 (50)
	Female	9 (50)
Occupation	Community pharmacists	6 (33.3)
	Pharmacy technicians	2 (11.1)
	Dispensing assistants	10 (55.6)
Working experience (years)	Less than 2 years	5 (27.7)
	2 to 3 years	10 (55.6)
	4 to 5 years	3 (16.7)

### STIs management practices

The themes derived from the study were knowledge of Standard Treatment guidelines (STGs) and STI management, attitude and perception towards the use of STGs and practice of the respondents in STIs management (Table 2).

**Table 2:** Thematic table on STIs management practices

Theme	Sub-theme
Knowledge on standard treatment guidelines (STGs) and the management of STIs	1. Most had adequate knowledge of STGs in STIs management 2. Others were unaware of STGs in STIs management
Attitude and perception towards the use of STGs	1. STGs are very important 2. Recommendations from the STGs are sometimes not effective 3. Syndromic treatment is ineffective
Providers’ practice of STIs management	1. STGs were consistently employed in conjunction with the recommended tests and antibiotics 2. Comfortable in discussing STIs related matters 3. Do not consider multiple infections

### Knowledge of STGs and the management of STIs

Most of the participants had adequate knowledge of STGs for managing STIs. Some participants explained what STGs are about and how they are used in managing STIs. They noted that STGs contained conditions, signs and symptoms and possible prescriptions or medications for treating them. Some, however argued that the recommendations from the STGs are not exhaustive as sometimes laboratory tests are necessary before treatment and that some medications not included in the STGs may be more effective. The following quotes present their views:

*The STG recommendations are okay, with uncomplicated STIs but is only complicated ones that is not okay. Mmm, for the uncomplicated ones I will say, syndromic management is the best, but for the complicated ones the person would have to go to the hospital for further investigations like the laboratory tests and stuff hence syndromic approach is not the best for that side. —*Dispensing assistant (R9)

*So, we usually use syndromic approach. And for gonorrhoea, usually I treat with Cefixime 400mg, a start dose. And with chlamydia usually Doxycycline because is cheap. Syphilis, errh, I usually go for Azithromycin because we cannot give injection here, so. *—Community pharmacist (R6)

“Yes, we have a protocol, we follow the standard treatment guidelines. Depending on the symptoms too, we let the patient sometimes go for laboratory test.” —Dispensing assistant (R14)

*“Yes, we have – we have a protocol. We use erh erh – STGs – the Standard Treatment Guidelines*

Erh, we often go with the antibiotics, mostly the broad spectrums: azithromycin, doxycycline, erm metronidazole. Those – those are the ones we use.”—Pharmacy technician (R5)

Few participants, nevertheless, were not aware of STGs for STI management. This was more typical of the dispensing assistants. They claimed to be ignorant about STGs and that they were not aware that STGs should be used to guide the diagnosis and treatment of illnesses, including STIs. A dispensing assistant stated, “*I have not heard of STGs*.”

### Attitude and perspectives of respondents towards use of Standard Treatment Guidelines

Participants expressed their views and perception towards the use of Standard Treatment Guidelines in STI management. This section explored the perception of the participants on STGs use in STIs management and found two main opinions, namely; STGs are very important and recommendations from the STGs are not effective sometimes. Some participants noted that STG is an essential tool in the management of STIs as it guides treatment choices and decisions based on accepted standards. They noted that without the guidelines, practitioners would have to practice trial and error, which can have detrimental effects on the patients’ health.

Nevertheless, some participants argued that in some cases, the treatment in the guidelines is not effective in treating STIs. They intimated that sometimes they gave a particular medication as indicated by the STG but after that the patient returned without their signs and symptoms being resolved. The poor management recommended by the guidelines has resulted in poor perception of the guide’s effectiveness in treating STIs among some of the participants. A community pharmacist (R5), stated;

*I think the STG should be reviewed often because there are cases of STIs when we refer to the STG, we give the medications to the people and then they come back. And then sometimes, you don’t know the duration for which the person has been carrying the STI so if you give the STG recommended treatment and then the person goes and come back with the same condition, it’s a problem. So, I think it needs to be reviewed often so that we can have an update of what is going on.* —Community pharmacist (R5)

Another community pharmacist (R4) opined;

*Personally, I have a lot of issues with the STG recommendations on the management of STIs, especially because of the syndromic approach being used. You see, personally I don’t like that approach but I mean so far as it works; patients are also relieved of their symptoms.* —Community pharmacist (R4)

“But I wish the STG will be updated every six months, because people who are resistant to antibiotics, you give the drugs to them and they wouldn’t even recover.”—Dispensing assistant (R15).

More so, some participants, argued that syndromic treatment is not good. They argued that it is important for the causative organism to be confirmed before initiating treatment, as a poor choice of antibiotics may contribute to antimicrobial resistance or treatment failure. They further noted that on this basis, they believed that syndromic treatment is not the best practice in STI management. Their views are presented below:

*Erh, syndromic management – erh, I think that that’s where the problem is because we are just using symptoms to manage the cases and um, organisms are like that, they change. So, I think if we should do the test – run the tests for the various infections before we start treatment. That will be the best.*—Pharmacy technician (R5)

*Personally, I have an issue because I am a firm believer of antimicrobial resistance, so I mean going by the syndromic approach, we are definitely going to increase, ehh... we are going to have increase in the antibiotic resistance but sometimes you are not sure, though we could see that anytime someone come with gonorrhea, there is a likelihood the person is having gonorrhea but not 100%. So therefore, if you introduce the person to another antibiotic, you turn to increase the risk of antimicrobial resistance. Personally, am not a believer of syndromic approach but I still practice it anyways due to the absence of lab or testing. *—Community pharmacist (R4)

### Practices of STIs management

Practice of STIs management was also explored. Three subthemes (two positive and one negative) on the practice of STIs management among community pharmacy staff emerged. The two positive practices were consistent use of STGs in conjunction with the recommended tests and antibiotics for treatment and comfortable in discussing STIs related topics with clients. The third, a negative practice was not considering multiple infections in treatment practice.

The majority adhered closely to the STGs proposed antibiotic regimen, while a few incorporated the recommended STG laboratory tests in conjunction with antibiotic treatment. Regarding participants’ strict use of STG, many of the participants explained that they always followed the guidelines. They explained that they diagnosed the symptoms as stated in the guidelines and conclude on a diagnosis based on the signs and symptoms, then prescribe the recommended antibiotics. They, thus, confirmed syndromic treatment. Their quotes are presented as follows:

*We usually use the STG and most of our workers believe that if someone comes with the normal STI, we do the syndromic management. So, they give Ciprofloxacin tablets, Metronidazole or Doxycycline, but then sometimes, they also give Azithromycin and then Ciprofloxacin, it depends on who is serving the person and they try to cover all basis. I think the STG is good, I think it is fine. —*Community pharmacist (R1)

*So, as I said we follow the STGs, so for gonorrhea and chlamydia, we just go with, emmm… we just go with either Ciprofloxacin 500mg stat or we can also go with Cefixime 400gram stat and Azithromycin 1 g stat. Syphilis we don’t usually manage them here, we usually inform them to go, for them to have their shot (Benzathine penicillin). *—Community pharmacist (R4)

Consequently, some participants relied on the results of the laboratory tests before providing the drug therapy recommended by the STG. They noted that sometimes they requested for some laboratory tests to be carried out before prescribing the recommended treatment.

“Yeah, that’s why we use the multiple treatment. Sometimes when they come to you, they haven’t done the test. So, we run the tests to see which of the infection it actually is.” —Pharmacy technician (R5)

Furthermore, most of the participants also felt comfortable in discussing STI-related issues with their clients however, some of the clients were very shy and failed to open up to the pharmacy staff for informed decisions to be made on the therapy.

“Yes I am comfortable discussing STI related matters with my patients, why not we do it paa (a lot).”—Dispensing assistant (R16)

*Yes, but they won't tell you what you want to know so you won't force further. There is different form of communication uh-huh, personal and stuff. So, if the person does not allow you to know them personally, you wouldn't force further.*—Pharmacy technician (R8)

“Oh yes, I am very comfortable, the clients are sometimes not comfortable but for me, I am very comfortable.”—Community pharmacist(R4)

A major treatment practice among the participants was not considering multiple infections. The participants always provided treatment for the suspected STI condition based on the symptoms and did not consider the presence of other infections. They noted that they only prescribed treatment for the most probable STI based on the signs and symptoms given by the client/patient. Consequently, after deciding the most likely condition based on these symptoms, they give the recommended antibiotics.

*Emm… Usually, I usually treat them based on the symptoms that they came with and the impressions that has been able to form so far, just manage them for and then you try to advise him/her on all the risky behaviours, multiple partners and if possible, we encourage them to come with their partners so that they are also treated.* —Community pharmacist (R4)

*When we investigate them and they are able to give us the real symptoms, and also tell us what they are going through, we can know the particular infection we are treating. So, I can say no to your question, we don't treat it as multiple infection, yeah. —*Dispensing assistant (R9)

## Discussion

This qualitative study investigated the practices of the community pharmacists and other pharmacy support staff on the management of STIs within the Ho municipality. The findings from this research showed that the community pharmacists as well as the other pharmacy staff had sufficient knowledge in the management of STIs as suggested by the national STGs. The participants also stated that the guidelines (STG) were very important and useful even though some of the recommendations were ineffective at times. The participants’ positive practices were the consistent use of the STG as a guide and comfortable when engaging in discussions pertaining to sexually transmitted infections (STIs) with the clients whiles the negative practice was not treating STIs as multiple infections.

This study showed that the pharmacy staff had adequate knowledge on the national Standard Treatment Guidelines and used the recommended treatments from the guidelines to make informed decisions on drug therapies. The participants also stated that they used the STG as their standard protocol even though a few of the participants who were dispensing assistants did not know what the STG was. This deficiency in knowledge could be due to insufficient training such as attendance of continuous professional programs and proper in-service training. Inadequate knowledge, deficient training, and a lack of specific protocols are the primary obstacles to providing sexual health and STI services in primary care settings such as the community pharmacy [22]. Comparable investigations conducted by Chesang et al. [23], Gauly et al. [24], Navarrete et al. [25] have documented that community pharmacists possess a commendable level of knowledge pertaining to the management of STIs. Additionally, these investigations elucidate that these community pharmacies constitute the primary point of contact for individuals seeking assistance with sexual and reproductive health concerns.

Healthcare providers in Ghana use the national STGs as a guide when diagnosing patients, administering drugs, and treating symptoms related to common diseases [26]. In addition, the consistent use of STGs in STIs management observed in this study is consistent with the WHO's position that syndromic management of STIs is the primary healthcare approach to STIs management [11]. Some participants stated that recommendations by the STG were not updated regularly considering the current trends of antimicrobial resistance hence there was fear of treatment failure and antibiotic resistance. The STG may be revised regularly to increase the health providers’ trust in the health care system and national guidelines as well as synchronizing it with global guidelines on managing STIs.

The use of STGs by the pharmacy staff in this study is in concordance with the management of STIs in most resource-limited settings as recommended by the WHO [11, 27]. Due to the unavailability or limited accessibility of laboratory diagnosis, syndromic management flowcharts are frequently used as the standard of care [11]. Even though laboratory tests are essential to the management of STIs, STGs serve as a benchmark to assure, to a great degree, the proper diagnosis as well as the correct prescribing of antibiotics for STIs. Consequently, the suggestions from the national STG have been developed to be employed by health professionals who offer STI prevention and control services at all levels of healthcare [27].

Some participants raised concerns about the syndromic approach to the management of STIs being presumed the benchmark in the community pharmacy setting due to the difficulty in getting access to laboratory tests to detect the presence of the likely organisms before initiating treatment on the same day. Most healthcare providers (HCPs) used syndromic management, even though many had no access to the treatment algorithm and STI guidelines. While appreciating the extra resource requirements for etiologic diagnosis, some HCPs would prefer this approach because it offers improved specificity in diagnosis, better management of recurrent infection, elimination of unnecessary medications, and monitoring for drug resistance [23]. In contrast, Chikwari et al. recommends we move away from syndromic management of STI with its low sensitivity to aetiological approaches, ideally incorporating point-of-care diagnostics that will facilitate high uptake of testing and high/timely treatment [28].

Some participants did not treat multiple infections, which is not a good practice because many infections could be missed and detrimental to the patient’s health. According to the STG, there is a difficulty in targeting the likely organism causing STI when using the syndromic approach hence it is necessary to prescribe antibiotics to cover several likely causes of the suspected infections that the patient presents to the community pharmacy [27]. This current study finding was not in agreement with the national guidelines. The possible reasons for this practice could be due to the cost of antibiotics, limited access or no access to laboratory tests and antibiotic resistance.

Majority of the participants said that they were comfortable discussing STI-related matters with their clients. Healthcare providers are enthusiastic about treating STIs, but they dislike discussing sexual matters with patients, particularly anal sex, contrary to the findings of this study, which revealed that the pharmacy staff were quite comfortable discussing sexually related issues [23].

The Pharmacy Act, 1994 (Act 489) in Ghana prohibits pharmacy assistants from selling and dispensing Class A/prescription-only medicines, including antibiotics, and Class B/pharmacy-only medicines [29]. This study showed that the dispensing assistants and pharmacy technicians dispense prescription medications directly to the patients and this is considered a bad practice. This could be due to lack of enforcement of the regulatory laws of practice, inadequate number of pharmacists and the high cost of employing more pharmacists.

### Limitations

Practice of STI management was self-reported which has the tendency of resulting in underreporting of negative practices and over reporting of positive practice behaviors. Nevertheless, participants were encouraged to be as honest as possible in their responses. This study is also limited to gonorrhoea, syphilis and chlamydia and no other STIs.

## Conclusion

The study showed that community pharmacists and other pharmacy staff had good level of awareness and knowledge of STGs and contribute significantly to the management of STIs employing the STGs, either syndromic treatment and/or using laboratory tests, to treat STIs. However, many of them have unfavorable views of the relevance of the national STG in STIs management. These come with concerns for ineffective therapy, treatment failures, STI complications and antimicrobial resistance. For efficiency and optimal outcomes, the National STI control programme should invest in improving the national STG to stay relevant to changing global recommendations and views of practitioners as well as training of healthcare providers, including community pharmacy practitioners. The pharmacies should also be encouraged to have diagnostic kits or work with laboratory facilities for testing common STIs to inform definitive therapy and improved outcomes.

### Supplementary Information

**Additional file 1.** Interview guide for community pharmacy staff.

## Data Availability

Data for the study is embedded in the manuscript as quotations.

## Abbreviations

AIDS: Acquired immunodeficiency syndrome
HCP: Healthcare provider
HIV: Human immunodeficiency virus
MOH: Ministry of Health
NACP: National AIDS Control Programme
PID: Pelvic inflammatory disease
STIs: Sexually transmitted infections
STG: Standard Treatment Guidelines
WAPTCA: West Africa Project to Combat AIDS and STD
