# Supplementary material for: Management of sexually transmitted infections: a qualitative assessment of community pharmacy practices in the Ho Municipality, Ghana
Source: J Pharm Policy Pract. 2023 Nov 10;16:140. doi: 10.1186/s40545-023-00650-0 (PMC10636839; doi:10.1186/s40545-023-00650-0)
Supplement: Supplementary file 1 — Additional file 1. Interview guide for community pharmacy staff. [file 40545_2023_650_MOESM1_ESM.docx]

**COMMON SEXUALLY TRANSMITTED DISEASES AND THEIR MANAGEMENT**

**INTERVIEW GUIDE FOR COMMUNITY PHARMACY STAFF**

**PART I**

1. **Demographic characteristics of the participants**

Age (years): ˂ 21 [ ] 21-30 [ ] 31-40 [ ] 41-50 [ ]

Sex: Male [ ] Female [ ]

Occupation: Community Pharmacist [ ] Dispensing assistant [ ]

Pharmacy Technician [ ]

Working experience (years): ˂ 2 [ ] 2-3 [ ] 4-5 [ ] ˃5 [ ]

**PART II**

1. **Knowledge and perception of the national Standard treatment guidelines in the management of STI**

- Does your facility have a protocol/guide for the management of STI?
- How are the various curable bacterial STIs (gonorrhea, chlamydia and syphilis) treated in this community pharmacy?
- What is your opinion on the Standard treatment guidelines (STG) recommendations for the management of STI (gonorrhea, syphilis and chlamydia)?

**PART III**

1. **STI management practices**

- Is syndromic management the best approach to the management of STIs?
- What investigations should be conducted before treatment begins?
- In the face of risky sexual behaviors, multiple sexual partners and a high rate of unprotected sex, would you prefer to treat patients with STI as multiple infections?
- Are you comfortable discussing STI-related matters with clients?
